# Correlation between Systemic Oxidative Stress and Intraocular Pressure Level

**DOI:** 10.1371/journal.pone.0133582

**Published:** 2015-07-17

**Authors:** Masaki Tanito, Sachiko Kaidzu, Yasuyuki Takai, Akihiro Ohira

**Affiliations:** 1 Division of Ophthalmology, Matsue Red Cross Hospital, Matsue, Shimane, Japan; 2 Department of Ophthalmology, Shimane University Faculty of Medicine, Izumo, Shimane, Japan; Casey Eye Institute, UNITED STATES

## Abstract

**Background:**

The involvement of local and systemic oxidative stress in intraocular pressure (IOP) elevation and optic nerve damage has been hypothesized in the pathogenesis of glaucoma. We reported previously that the level of systemic antioxidative capacity is lower in patients with open-angle glaucoma than controls without glaucoma. Here, we assessed the correlation between IOP and systemic levels of prooxidants and antioxidants by analyzing the blood biochemistry in patients with glaucoma.

**Methods:**

Peripheral blood samples were collected from Japanese patients with primary open-angle glaucoma (n = 206), exfoliation syndrome (n = 199), and controls (n = 126). Serum levels of lipid peroxides, ferric-reducing activity, and thiol antioxidant activity were measured by diacron reactive oxygen metabolite (dROM), biological antioxidant potential (BAP), and sulfhydryl (SH) tests, respectively, using a free radical analyzer. To test the possible effect of oxidative stress on IOP levels, the patients were classified into one of four groups (Q1, Q2, Q3, and Q4, with Q1 having the lowest IOP) based on the quartile value of IOP. For this classification, the known highest IOP value in both the right and left eyes was regarded as each subject’s IOP. For comparisons among the IOP groups, the differences were calculated using one-way analysis of variance followed by post-hoc unpaired t-tests. To adjust for differences in demographic characteristic distributions, the dROM, BAP, and SH test values were compared among the IOP groups using multiple logistic regression analysis; the odds ratio (OR) of each variable was calculated with the Q1 group as the reference.

**Results:**

The dROM and the SH levels did not differ significantly (p = 0.6704 and p = 0.6376, respectively) among the four IOP groups. The BAP levels differed significantly (p = 0.0115) among the four IOP groups; the value was significantly lower in the Q4 group (1,932 μmol/L) compared with the Q1 (2,023 μmol/L, p = 0.0042) and Q2 (2,003 μmol/L, p = 0.0302) groups and significantly lower in the Q3 group (1,948 μmol/L) than the Q1 (p = 0.0174) group. After adjustment for differences in various demographic characteristics, lower BAP values were significantly associated with the classification into higher IOP groups (Q3 group, p = 0.0261 and OR = 0.06/range; Q4 group, p = 0.0018 and OR = 0.04/range). The dROM and SH values did not reach significance in any comparisons.

**Conclusions:**

Lower systemic antioxidant capacity measured by ferric-reducing activity is involved in the pathogenesis of open-angle glaucoma via its roles in IOP elevation.

## Introduction

Glaucoma, which is characterized by progressive “glaucomatous” optic neuropathy and visual field loss, is a leading cause of irreversible blindness worldwide [1,2], including Japan [3]. Retinal ganglion cell (RGC) death resulting from apoptosis and RGC axon loss leads to glaucomatous optic neuropathy, in which elevated intraocular pressure (IOP) is the primary risk factor [2]. In open-angle glaucoma (OAG) including primary OAG (POAG) and glaucoma secondary to pseudoexfoliation syndrome (EX), the IOP increases as the result of reduced aqueous humor outflow at the trabecular meshwork (TM) [4]. This results from dysfunctional TM cells and consequent changes in the amount and quality of the extracellular matrix (ECM) in the TM [5]. Clinical and experimental studies have reported that oxidative stress and/or inflammation associated with TM cell dysfunction and aqueous outflow resistance increase [6–10].

Oxidative stress is induced through formation of multiple reactive oxygen species including superoxide, hydrogen peroxide, and hydroxyl radicals that can initiate and propagate free radicals. The net oxidative burden between the prooxidant and antioxidant systems is oxidative stress, which damages cellular and tissue macromolecules such as lipids, proteins, and nucleic acids and results in cellular and tissue dysfunction and cellular death. The systemic status of redox (reduction/oxidation) in glaucoma has been the subject of increasing interest following identification of the circulating autoantibodies against antioxidative stress enzymes and chaperone molecules glutathione S-transferase [11] and heat shock proteins [12,13] in the serum of patients with glaucoma. A number of studies have reported on this topic [14–27]. We assessed the systemic redox status of glaucoma more comprehensively by including a larger number of subjects than previously studied and by simultaneously testing the oxidative and antioxidative status. We identified significantly lower systemic antioxidant capacity level in subjects with POAG and EX compared with controls [28].

Although a few studies have evaluated a correlation between glaucoma severity (i.e., IOP or visual field damage) and ocular [29,30] or systemic [22] levels of oxidative stress in humans, the role of systemic oxidative stress in glaucoma pathogenesis remains largely unknown. In the current study, we statistically analyzed our previously established dataset (S1 Table) [28] to assess a possible correlation between IOP and systemic levels of prooxidants and antioxidants.

## Subjects and Methods

### Subjects

The study adhered to the tenets of the Declaration of Helsinki. The institutional review boards of Shimane University Hospital and Iinan Hospital, Shimane, Japan reviewed and approved the research. All subjects provided written informed consent. A total of 531 Japanese subjects with OAG (POAG or EX) and subjects without glaucoma were recruited consecutively at both hospitals. All subjects underwent ophthalmologic examinations including measurements of the best-corrected visual acuity (VA) and IOP by Goldmann applanation tonometry and slit-lamp, gonioscopic, and funduscopic examinations through dilated pupils. The diagnosis and demographics of each group were described previously [28]. Briefly, POAG was diagnosed based on open iridocorneal angles bilaterally, the characteristic appearance of glaucomatous optic neuropathy such as enlargement of the optic disc cup or focal thinning of the neuroretinal rim, corresponding visual field defects identified using the Humphrey Visual Field Analyzer (Carl Zeiss Meditec, Dublin, CA) in at least one eye, and no evidence of secondary glaucoma bilaterally. EX was diagnosed based on an open iridocorneal angle and characteristic pseudoexfoliation material deposits on the anterior capsule and/or pupillary margin in at least one eye; subjects without glaucoma had a corrected VA of 0.7 or better measured in both eyes using a decimal VA chart and no glaucomatous optic neuropathy or history of IOP of 21 mmHg or higher.

### Recording Clinical Parameters and Collecting Blood Samples

To avoid the possible confounding effect of systemic diseases [31–33], the subjects were questioned about a history of severe systemic diseases during an interview before entry into the study. These diseases included acute brain infarction and hemorrhage, systemic neurologic diseases, cardiac diseases requiring catheter placement or surgery, cardiac failure and other systemic diseases affecting the subjects’ physical activity, lung diseases causing dyspnea, chronic and acute hepatitis requiring interferon therapy, liver cirrhosis, renal failure requiring hemodialysis, autoimmune diseases requiring systemic steroids and other immunosuppressive therapies, severe anemia requiring blood transfusions, major visceral surgery, malignancies, and severe hypertension causing cardiac and kidney failure, and severe diabetes requiring insulin therapy. In addition to a history of severe systemic diseases, to adjust for the possible confounding effects of other factors such as differences in nutrition, blood pressure, blood glucose, and smoking habits [34–36], the presence or absence of diabetes, current smoking, time since the last meal, and systolic (SBP) and diastolic (DBP) blood pressures and pulse rate were recorded before blood samples were collected. The highest known IOP measured on the day of sample collection or previously measured IOP recorded in the medical charts also was collected. All subjects were classified into four groups (Q1, Q2, Q3, and Q4, with Q1 having the lowest IOP) based on the quartile value of IOP. For this classification, the higher IOP between both eyes was regarded as each subject’s IOP. Venous blood specimens were collected from the antecubital vein into evacuated tubes. Serum samples obtained by centrifugation of the collected venous blood were stored at 4˚C until oxidative stress measurements. During all handling procedures, including transportation from the clinical setting to the laboratory and centrifugation, the temperature was maintained at 4˚C.

### Oxidative Stress Measurements

All blood analyses were performed using a free radical analyzer system (FREE Carpe Diem, Wismerll Company Ltd., Tokyo, Japan) that included a spectrophotometric device reader and a thermostatically regulated mini-centrifuge; the measurement kits were optimized to the FREE Carpe Diem System, according to the manufacturer’s instructions. Based on the manufacturer’s recommendation, all analyses were performed within 48 hours of venous blood collection to avoid falsely high or low results. To analyze the serum levels of reactive oxygen metabolites, antioxidant capacity, and thiol-antioxidant capacity, diacron reactive oxygen metabolite (dROM), biological antioxidant potential (BAP), and sulfhydryl (SH) tests were performed, respectively. The results of dROM testing were expressed in arbitrary units (U. Carr), one unit of which corresponds to 0.8 mg/L of hydrogen peroxide [34,37]; the results of the BAP test were expressed in μmol/L of the reduced ferric ions; the results of the SH test were expressed as μmol/L of the SH groups. A comparison of the measured levels of oxidative stress between the non-glaucoma and glaucoma groups were reported previously [28].

The dROM test reflects the amount of organic hydroperoxides that is related to the free radicals from which they are formed. When the samples are dissolved in an acidic buffer, the hydroperoxides react with the transition metal (mainly iron) ions liberated from the proteins in the acidic medium and are converted to alkoxy and peroxy radicals. These newly formed radicals oxidize an additive aromatic amine (*N*,*N*-diethyl-*para*-phenylen-diamine) and cause formation of a relatively stable colored cation radical that is spectrophotometrically detectable at 505 nm [34,37]. The results are expressed in arbitrary units (U. Carr), one unit of which corresponds to 0.8 mg/L of hydrogen peroxide [34,37].

The BAP test provides an estimate of the global antioxidant capacity of blood plasma, measured as its reducing potential against ferric ions. When the sample is added to the colored solution obtained by mixing a ferric chloride solution with a thiocyanate derivative solution, decoloration results. The intensity of the decoloration is spectrophotometrically detectable at 505 nm and is proportional to the ability of plasma to reduce ferric ions [35,38]. The results are expressed in μmol/L of the reduced ferric ions.

The SH test provides an estimate of the total thiol groups in the biologic samples, using a modified Ellman method [39,40]. When the sample is added to the solution, SH groups in the sample react with 5,5-dithiobis-2-nitrobenzoic acid, which is followed by development of a stained complex that is spectrophotometrically detectable at 405 nm and is proportional to their concentration according to the Beer-Lambert law [35,37]. The results are expressed as μmol/L of the SH groups.

### Statistical Analysis

The data are expressed as means ± standard deviations and analyzed using JMP version 10.02 statistical software (SAS Institute, Inc., Cary, NC). For comparisons among the four IOP groups, the differences in continuous data (age, SBP, DBP, pulse rate, time from the last meal, and dROM, BAP, and SH tests) were calculated using one-way analysis of variance followed by post-hoc unpaired t-tests. The differences in categorical data (sex, diabetes, and current smoking) were calculated using the Cochran-Armitage test for trend. To adjust for differences in demographic characteristic distributions among the IOP groups, the values of the dROM, BAP, and SH tests were compared between the IOP groups using multiple logistic regression analysis; the odds ratio of each variable was calculated with the Q1 group as the reference. P values of less than 0.05 were considered statistically significant.

## Results

The subject demographic data, including age, sex, SBP, DBP, pulse rate, time from the last meal, diabetes, and smoking status, are summarized in Table 1. The comparisons among the IOP groups showed that more men (p<0.0001) and subjects with glaucoma (p<0.0001) were classified into higher IOP groups than lower IOP groups; the SBP (p = 0.0133), DBP (p = 0.0037), and pulse rate (p = 0.0007) were higher in groups with higher IOP than in groups with lower IOP. The other parameters did not differ significantly among the four IOP groups.

**Table 1:** Demographic subject data.

	Q1	Q2	Q3	Q4	p value
range (mmHg)	Low- ≤16	>16 - ≤19	>19 - ≤26	≥26—High	
n	136	131	133	131	
Age (years)					
Mean ± SD	72.9 ± 11.6	73.4 ± 9.6	72.8 ± 10.8	74.5 ± 10.5	0.5530a
range	23–93	25–96	38–90	24–94	
Sex					
Men, n (%)	47 (35)	48 (37)	55 (41)	81 (62)	<0.0001b
Women, n (%)	89 (65)	83 (63)	78 (59)	50 (38)	
Disease (n (%))					
non-glaucoma	61 (45)	51 (39)	14 (11)	0 (0)	<0.0001b
glaucoma	75 (55)	81 (69)	119 (89)	131 (100)	
SBP (mmHg)					
Mean ± SD	138.4 ± 21.8	138.5 ± 20.1	136.7 ± 17.0	144.3 ± 21.4	0.0133a
range	85–203	94–223	85–173	101–211	
				vs Q1, p = 0.0165c	
				vs Q2, p = 0.0188c	
				vs Q3, p = 0.0022c	
DBP (mmHg)					
Mean ± SD	74.1 ± 11.5	74.4 ± 11.8	76.4 ± 12.7	79.2 ± 14.4	0.0037a
range	45–100	46–119	47–123	51–133	
				vs Q1, p = 0.0010c	
				vs Q2, p = 0.0023c	
Pulse rate (/minute)					
Mean ± SD	74.4 ± 13.7	71.3 ± 11.2	76.9 ± 12.7	77.1 ± 14.5	0.0007a
range	42–120	46–114	55–131	48–128	
			vs Q2, p = 0.0004c	vs Q2, p = 0.0003c	
Duration from last meal (h)					
Mean ± SD	3.8 ± 2.4	3.9 ± 1.8	3.8 ± 2.2	3.9 ± 4.0	0.1994a
range	1–18	1.5–16	1–19	0.5–14	
Diabetes					0.1315b
Yes, n (%)	22 (16)	20 (15)	30 (23)	28 (21)	
No, n (%)	114 (84)	111 (85)	103 (77)	103 (79)	
Current smoking habit					0.0657b
Yes, n (%)	8 (6)	11 (8)	13 (10)	16 (12)	
No, n (%)	128 (94)	120 (92)	119 (90)	115 (88)	

The p values were calculated using the one-way ANOVA (a) or Cochran-Armitage test for trend (b) among the Q1-4 groups or were calculated using the unpaired t-test (c) between each pair of indicated groups. SBP, systolic blood pressure; and DBP, diastolic blood pressure.

The measured levels of oxidative and antioxidative parameters in the serum of the subjects are shown in Table 2. The dROM levels (p = 0.6704), a measure of oxidative status, and the SH level (p = 0.6376), a measure of thiol-mediated antioxidative status, did not differ significantly among the four IOP groups. The BAP levels, a measure of antioxidative status, differed significantly among the four IOP groups (p = 0.0115); the value was significantly lower in the Q4 (1,932 μmol/L) group, the highest IOP group, than in the Q1 (2,025 μmol/L, p = 0.0042) and Q2 (2,003 μmol/L, p = 0.0302) groups and in the Q3 (1,948 μmol/L) group, the group with the second highest IOP, compared with the Q1 group (p = 0.0174).

**Table 2:** dROM, BAP, and SH values.

	Q1	Q2	Q3	Q4	p value
dROMs test (U. Carr)					
Mean ± SD	349.9 ± 64.6	351.8 ± 65.6	358.5 ± 57.3	356.3 ± 67.0	0.6704a
range	201–511	102–555	219–506	202–551	
BAP test (μmol/L)					
Mean ± SD	2024.6 ± 249.9	2002.5 ± 296.7	1948.0 ± 265.5	1931.7 ± 239.0	0.0115a
range	1218.1–2722.6	1241.5–2857	412.7–2520	1154.6–2560.2	
			vs Q1, p = 0.0174b	vs Q1, p = 0.0042b	
				vs Q2, p = 0.0302b	
SH test (μmol/L)					
Mean ± SD	609.0 ± 105.1	600.0 ± 100.1	608.7 ± 89.9	596.4 ± 90.7	0.6376a
range	257–864	288–847	389–884	328–833	

The p values were calculated using the one-way analysis of variance (a) among the Q1-4 groups or were calculated using the unpaired t-test (b) between each pair of indicated groups. dROM, diacron reactive oxygen metabolites; BAP, biological antioxidant potential; and SH, sulfhydryl.

Since several demographic parameters differed among the IOP groups (Table 1), multivariate logistic regression models were used to adjust for any possible confounding effects of these parameters on the difference in oxidative and antioxidative status among the IOP groups (Table 3). The models did not reach significance between the Q1 and Q2 groups (p = 0.7342) but did reach significance between the Q1 and Q3 groups (p<0.0001) and the Q1 and Q4 groups (p<0.0001). In both models, lower BAP values were associated significantly with the classification into the higher IOP groups (p = 0.0261 and OR = 0.06/range for Q3 group, and p = 0.0018 and OR = 0.04/range for Q4 group); although the higher dROM values may be related to the classification into the Q4 group (p = 0.1002, OR = 3.97/range), the dROM and SH values did not reach significance in any comparisons. Other than BAP value, we found that male sex (p = 0.0111 and OR = 2.81 for Q4), glaucoma (p<0.0001 and OR = 7.64 for Q3 and p<0.0001 and OR = 1.42x10^11^ for Q4), lower SBP (p = 0.0193 and OR = 0.09/range for Q3) and higher DBP (p = 0.0292 and OR = 12.05 for Q3) were related to the classification into higher IOP groups.

**Table 3:** Multivariate logistic regression analyses.

	Q1	Q2	Q3	Q4
Entire model				
p value	—	0.7342	<0.0001	<0.0001
Age (years)				
p value	—	0.7012	0.5806	0.4297
OR (95% CI)/range	1	1.51 (0.18–12.95)	1.82 (0.22–15.61)	2.81 (0.21–36.22)
Sex				
p value	—	0.8988	0.8406	0.0111
OR (95% CI) men/women	1	0.96 (0.55–1.69)	0.94 (0.50–1.75)	2.35 (1.22–4.67)
Disease (n (%))				
p value	—	0.3478	<0.0001	<0.0001
OR (95% CI) glaucoma/non-glaucoma	1	1.28 (0.77–2.15)	7.64 (3.94–15.73)	1.42x10e+11 (50.95-)
SBP (mmHg)				
p value	—	0.6584	0.0193	0.2153
OR (95% CI) /range	1	0.62 (0.07–5.17)	0.09 (0.01–0.68)	0.25 (0.03–2.24)
DBP (mmHg)				
p value	—	0.3013	0.0292	0.1377
OR (95% CI) /range	1	3.06 (0.37–26.27)	12.05 (1.28–129.62)	8.77 (0.50–167.67)
Pulse rate (/min.)				
p value	—	0.0202	0.4256	0.3495
OR (95% CI) /range	1	0.14 (0.03–0.74)	2.17 (0.33–14.95)	2.66 (0.34–21.60)
Duration from last meal (hours)				
p value	—	0.5100	0.6037	0.7982
OR (95% CI) /range	1	1.96 (0.26–15.33)	0.56 (0.05–4.85)	1.59 (0.05–66.07)
Diabetes				
p value	—	0.8595	0.2153	0.7603
OR (95% CI) y/n	1	0.94 (0.47–1.87)	1.56 (0.77–3.21)	1.14 (0.51–2.63)
Current smoking habit				
p value	—	0.2782	0.1803	0.1207
OR (95% CI) y/n	1	1.79 (0.63–5.40)	2.25 (0.69–7.90)	2.86 (0.77–14.00)
dROM test (U. Carr)				
p value	—	0.9337	0.3642	0.1002
OR (95% CI) /range	1	1.08 (0.28–6.43)	1.91 (0.47–7.88)	3.97 (0.77–21.50)
BAP test (μmol/L)				
p value	—	0.4528	0.0261	0.0018
OR (95% CI) /range	1	0.56 (0.12–2.53)	0.06 (0.00–0.72)	0.04 (0.01–0.31)
SH test (μmol/L)				
p value	—	0.5839	0.5575	0.9837
OR (95% CI) /range	1	0.63 (0.12–3.32)	1.82 (0.25–13.70)	0.98 (0.12–8.26)

To adjust the difference in background characteristics among the IOP groups, *p* values were calculated using multivariate logistic regression analyses to compare the Q1 and the Q2, Q3, and Q4 groups. For calculation of the odds ratios, the Q1 group was the reference.

SBP, systolic blood pressure; DBP, diastolic blood pressure; ROM, diacron reactive oxygen metabolites; BAP, biological antioxidant potential; SH, sulfhydryl; OR, odds ratio; CI, confidence interval.

## Discussion

Previous studies have assessed the systemic redox status in glaucoma [14–27]. The current study, which included 531 subjects, is the largest such study. Several previous studies have reported lower systemic levels of antioxidants or antioxidative stress capacity in glaucoma [15,18,20,21,23,28]. Other studies have tested simultaneously the status of systemic and local redox [16,17,22,24]. Yagci et al. found increased protein carbonylation, which is a measure of protein oxidation, in aqueous and serum samples from EX compared with controls [16]. Koliakos et al. identified significantly lower levels of antioxidative stress enzyme catalase activities in aqueous and serum samples from EX compared with controls [17]. Nucci et al. described significantly lower total antioxidant capacity in aqueous humor and blood samples from patients with POAG compared with controls [24]. Sorkhabi et al. found a correlation between a higher aqueous humor 8-hydroxy-2’-deoxyguanosine (8-OHdG) level, a marker of oxidative stress-induced DNA damage, and a higher 8-OHdG level and lower antioxidant capacity levels in serum samples from patients with glaucoma [22]. Thus, the systemic antioxidant capacity can reflect the local ocular redox status.

Experimentally, various oxidative stresses induce RGC death [41,42], and free-radical scavengers prevent glaucomatous tissue injury such as glutamate- and IOP-induced RGC death [43,44] and tumor necrosis factor α-induced axonal injury [45]. Hydrogen peroxide treatment affects the cytoskeletal structure and cell-matrix interactions in TM cells [46]; depletion of glutathione and treatment with hydrogen peroxide decrease the TM outflow facility [47]. Increased 8-OHdG levels in human TM specimens are associated with higher IOP [9] and more severe visual field loss [29,30] in OAG; increased aqueous humor oxidative stress is associated with higher IOP in patients with EX [48]. Considering all those results, decreased systemic antioxidant capacity may be involved in glaucomatous TM and/or neuronal damage due to the local inadequate defense against oxidative stress. In the current study, univariate (Table 2) and multivariate (Table 3) analyses indicated that the BAP level, which indicates the total antioxidative stress activity, was lower in groups with higher IOP levels. Based on the multivariate regression analyses ORs, compared with the subject with the highest BAP value, the subject with the lowest BAP value has 17 and 25 times higher chances of being classified into the second highest and the highest IOP groups, respectively. Accordingly, the current results suggested that decreased antioxidant capacity is related more closely to IOP elevation than neuronal damage. Previous studies have assessed correlations between the systemic status of oxidative stress and clinical parameters of glaucoma. Engin and colleagues found a significant association between the systemic vitamin E level and the clinical parameters of glaucoma, i.e., IOP, changes in the optic nerve head, and visual field sensitivity [20]. The current study added to the body of evidence that systemic redox status affects IOP level. The analyses regarding the correlations between BAP values and severity indices of glaucoma such as visual field, and optic disc morphology using the data obtained in this study would be interest, and should be tested in the near future.

We did not find a significant association between the dROM level and IOP; in contrast, several studies have reported higher systemic oxidation levels in glaucoma [14,18,20,23]. The current study included eyes with mild cataract with a VA of 0.7 or better in the control group; however, most previous studies have included control subjects who did not have cataracts. Because the presence of a cataract might be related to increased systemic oxidation [49,50], including patients with a cataract may weaken the statistical power to detect the roles of dROM in IOP. Alternatively, if the absence of a correlation between systemic dROM levels and IOP is real, glaucomatous damage in the TM may be caused by a local increase in oxidative stress due to local compensation for systemic oxidative stress by systemic reduction of antioxidant capacity in each individual.

The current study found no significant association between SH and IOP. The glutathione and thioredoxin systems are thought to be major thiol-mediated redox systems in humans, although the plasma glutathione level is 100 to 1,000 times higher than that of thioredoxin [51,52]. Gherghel and coworkers reported a negative correlation between age and total glutathione levels in red blood cells with no significant difference in the total glutathione level between patients with glaucoma and controls [15]. Thus, the SH level found in the current study may correspond mainly to the total glutathione level; however, this requires clarification. Previous studies have reported that thioredoxin system dysregulation may be a factor in the pathogenesis of glaucoma [43,44,53–55]. Measurement of the thioredoxin level separately from other thiol groups should be of interest.

We found that male sex, lower SBP, and higher DBP can be associated with classification into higher IOP groups. Multiple population-based studies have reported conflicting results regarding the risk for glaucoma development between men and women [56] as well as conflicting results regarding the risk of glaucoma development between low and high blood pressures [57]. The roles of gender and BP on IOP remain inconclusive and require further study. For analyzing the correlation between systemic oxidative stress and IOP levels, we had chosen either one eye from each subject; we had used the known highest IOP in both eyes as the criteria. Because of the exclusion of eyes with the lower known highest IOP, it is possible that the analyses of this study might overestimate the roles of oxidative stresses in IOP elevation. However, as discussed previously, most of reported evidences suggested that systemic and local oxidative stresses more likely correlate with higher IOP rather than lower IOP, thus we believe that the selection method we used is reasonable for aim of this study. In this study, one of the major determinants of IOP level, i.e., central corneal thickness, was not included in the analyses. To our best knowledge, corneal thickness itself is not the major determinant of systemic oxidative stresses, and vice versa. Accordingly, it seems that inclusion of central corneal thickness does not likely change our observation in this study, but requires a clarification.

Based on the results of the current comprehensive large-scale study, we concluded that lower systemic antioxidant capacity measured by ferric-reducing activity is involved in the pathogenesis of OAG via its roles in IOP elevation.

## Supporting Information

S1 Table Dataset underlying the findings described in this manuscript. (PDF)
