# Supplementary material for: Correlation between Systemic Oxidative Stress and Intraocular Pressure Level
Source: PLoS One. 2015 Jul 17;10(7):e0133582. doi: 10.1371/journal.pone.0133582 (PMC4506090; doi:10.1371/journal.pone.0133582)
Supplement: S1 Table — (PDF) [file pone.0133582.s001.pdf]

Table S1. Dataset

Age	Sex	DISEASE	MEAL	SBP	DBP	PULSE	DM	SMOKE	dROM	BAP	SH	IOP
73	M	GLA	3	151	87	112	n	n	490	1813.6	653	27
63	F	GLA	4	166	93	84	y	n	285	1850.1	640	58
85	F	GLA	5	122	65	68	n	n	368	2019.4	514	38
72	M	GLA	5	159	91	94	y	y	462	1521.8	577	20
74	F	GLA	6	134	51	90	n	n	441	1547.0	669	39
76	M	GLA	6	119	68	75	y	n	311	1708.5	743	35
52	M	GLA	2.5	151	106	110	n	y	448	990.1	593	22
77	M	GLA	3	157	100	94	n	n	336	1892.8	637	47
67	F	GLA	2	128	79	58	n	n	431	1737.0	582	17
71	F	GLA	2.5	136	74	83	n	n	302	2060.9	652	32
74	M	GLA	2	174	94	75	n	n	261	1798.6	626	18
79	F	GLA	2	141	65	81	y	n	304	2007.5	601	24
77	F	CT	4	126	68	72	n	n	396	2050.6	568	19
76	M	GLA	4	190	102	89	n	y	470	1628.0	546	39
65	M	GLA	4.5	211	133	92	n	n	309	1820.5	553	42
85	F	GLA	3.5	138	84	110	n	n	221	1516.6	588	40
68	M	CT	5	159	100	83	n	y	301	2072.7	639	20
50	F	GLA	2.5	140	87	73	n	n	274	2121.6	564	14
80	M	GLA	2.5	142	81	72	n	n	339	1994.9	567	19
73	F	GLA	2	172	93	94	n	n	474	1724.4	636	15
71	M	CT	3.5	120	57	67	n	n	254	1655.5	690	16
42	F	GLA	2	102	60	67	n	n	336	1827.8	630	23
59	F	CT	16	223	119	97	n	n	294	2058.6	728	18
61	F	GLA	3.5	121	71	94	n	n	281	1546.8	708	14
38	F	GLA	4	138	84	83	n	n	290	1904.4	692	21
79	M	GLA	3	164	86	86	y	n	334	1881.4	684	32
61	M	GLA	6.5	139	79	64	y	y	381	1928.4	620	52
87	M	GLA	2	164	81	81	n	n	308	2029.0	551	16
71	M	GLA	1	105	56	73	y	n	272	1452.8	681	34
82	M	GLA	2.5	167	96	100	n	n	412	1993.8	724	28
60	M	GLA	13	138	92	67	n	n	314	2013.3	807	22
60	F	GLA	4	134	65	87	n	n	310	1597.5	673	29
84	F	GLA	3.5	142	65	78	n	n	439	1944.0	640	52
42	F	GLA	4.5	109	71	84	n	n	255	1926.3	726	14
70	M	GLA	5	123	73	91	y	y	327	1654.5	551	21.6
68	M	CT	5.5	165	97	71	n	n	413	1801.8	596	20
78	M	GLA	5	146	71	109	y	n	287	1598.6	411	24
82	M	CT	5.5	143	81	82	n	n	309	1541.5	534	10
67	F	CT	5	136	69	87	n	n	344	1819.2	752	19
53	F	CT	6	159	84	74	n	n	401	1777.7	682	17
79	M	GLA	7.5	162	67	67	n	n	338	1354.5	541	38
76	F	GLA	6.5	125	75	104	n	n	379	1649.9	636	49
75	M	GLA	3	106	50	62	n	n	102	1572.2	390	17
70	M	GLA	4.5	125	71	71	y	n	296	1891.7	675	16
73	M	GLA	6	182	99	56	n	n	528	1262.0	606	19
86	F	GLA	5	133	74	84	n	n	337	1930.9	538	17
82	F	GLA	6	131	87	101	n	n	484	1834.5	475	56
75	F	GLA	2	151	69	92	n	n	372	2103.6	608	17
60	F	CT	3	139	88	79	n	n	388	2082.3	758	15
58	M	GLA	5	147	99	85	y	y	282	2038.1	777	37
81	F	GLA	4	150	92	66	n	n	336	2135.9	714	30
66	M	GLA	5	159	101	58	n	y	345	1806.8	686	60
79	M	GLA	3.5	141	87	104	n	n	436	1988.1	614	21
66	F	GLA	5.5	155	94	69	n	n	273	1659.0	724	55
82	F	GLA	5.5	127	74	80	n	n	551	1990.8	530	28
65	M	CT	5	117	65	83	n	n	302	1808.0	690	16
73	M	GLA	5	157	65	72	y	n	375	1866.4	688	27
44	F	GLA	4	105	63	64	n	n	378	2185.8	686	22
66	M	GLA	4	130	71	86	n	n	329	412.7	719	25
63	M	GLA	4	156	93	95	n	n	350	1861.5	630	50
62	M	CT	9	151	95	85	n	n	268	2659.5	577	17
84	M	GLA	4	153	76	96	y	n	423	2560.2	387	56
61	F	CT	6	146	97	73	n	n	376	2602.7	398	17
66	M	CT	7	126	85	73	n	n	352	2464.1	427	19
80	M	CT	6	153	76	78	n	n	306	2710.0	320	19
72	F	GLA	1.5	118	78	71	n	n	290	2339.7	414	19
77	M	CT	1	145	81	84	y	n	219	1799.9	389	19.3
72	F	GLA	3	121	71	68	y	n	337	2302.5	847	19
71	F	CT	16	134	76	87	n	n	332	2097.4	686	15.7
81	F	GLA	5	140	77	82	n	n	359	1999.7	608	47
87	F	GLA	5	164	77	82	n	n	344	2145.7	562	24
74	F	GLA	6	130	86	57	n	n	331	2074.6	648	30
56	M	CT	2	146	79	102	n	y	310	2346.1	737	16
76	M	GLA	3	135	83	80	n	n	289	1818.2	645	14
83	F	GLA	3.5	142	85	85	n	n	309	2188.9	572	20
61	M	GLA	3	148	92	63	y	n	378	1649.1	396	50
65	M	GLA	3	138	72	81	n	y	387	2024.1	474	26
60	M	GLA	5	140	73	91	y	n	406	1870.8	476	29
58	F	GLA	2	97	61	99	n	n	384	1927.1	396	21
49	M	GLA	3	140	94	87	n	n	278	1556.2	580	28
63	F	GLA	3	182	98	128	n	n	370	2028.0	556	39
78	M	GLA	3	126	81	73	n	n	342	2192.8	517	26
78	M	GLA	3	131	71	72	n	n	395	2015.6	558	28
61	M	GLA	3	110	64	60	n	y	358	2196.2	580	52
74	F	GLA	3	190	80	84	y	n	320	2000.2	596	27
64	F	GLA	5	137	91	102	n	n	347	2071.2	589	13
89	F	GLA	5	140	90	89	y	n	343	2293.7	486	42
74	M	GLA	3	125	64	80	n	n	318	1967.6	523	18
71	M	GLA	5	124	74	70	n	n	392	2183.0	790	33

58	M	GLA	4	142	85	68	n	n	386	1971.6	766	20
73	M	GLA	4	191	110	74	n	n	299	2112.5	727	30
59	M	GLA	5	172	103	78	n	n	333	1749.8	563	45
65	F	GLA	2.5	124	80	90	n	n	450	2077.5	692	28
81	F	GLA	3	137	74	81	n	n	324	1852.1	515	30
84	F	CT	5	127	78	67	n	n	391	2154.8	362	11
75	M	GLA	6	153	88	98	y	n	318	2151.9	499	25
81	M	GLA	6	138	96	72	n	y	359	2149.8	615	24
57	M	GLA	3	131	82	70	n	n	380	1459.1	606	25
50	F	GLA	4	119	87	93	n	n	365	2312.3	576	56
78	M	GLA	4	146	81	131	y	n	329	1878.4	439	23
74	F	GLA	2.5	146	58	79	n	n	265	2061.3	579	27
94	F	GLA	3	123	89	69	n	n	347	1728.5	543	34
80	F	GLA	2.5	129	77	83	n	n	362	1980.3	678	27
85	M	GLA	4	126	69	83	n	n	360	1961.7	497	39
74	F	GLA	6.5	130	84	103	n	n	505	2252.3	481	29
69	M	GLA	5	183	69	66	n	y	430	1966.2	520	43
67	F	CT	7	126	85	86	n	n	303	2113.9	542	15
78	M	GLA	8	134	70	108	n	n	258	2353.5	463	14
62	M	CT	2	137	83	85	n	n	350	2047.0	712	17
63	M	GLA	3.5	130	67	63	y	y	318	2130.1	725	22
64	F	GLA	3.5	126	75	82	n	n	402	1851.8	753	13
73	F	GLA	2	145	63	73	n	n	239	2143.0	629	14
68	F	CT	2	124	82	77	n	n	353	2246.0	689	14.3
58	F	GLA	3	130	88	70	n	n	379	2149.2	657	20
78	M	GLA	2	159	79	61	n	n	281	1897.6	583	35
74	F	GLA	2.5	150	78	78	y	n	280	2398.2	633	18
76	F	GLA	3	138	87	66	n	n	337	2129.0	613	17
81	F	GLA	4.5	149	79	80	n	n	345	2154.9	430	30
89	F	GLA	4	203	85	120	y	n	312	2381.0	760	13.7
54	M	GLA	4.5	137	83	83	n	y	370	1974.6	665	17
49	M	CT	2.5	139	90	74	n	n	340	2131.1	714	20
54	M	GLA	3.5	137	77	73	n	y	280	1617.7	642	17
76	F	GLA	3	108	60	69	n	n	314	1757.5	588	18
82	F	GLA	5	167	87	85	n	n	440	1774.9	559	13
72	M	GLA	4	163	91	72	y	n	311	2161.5	657	34
82	F	GLA	3.5	187	93	114	n	n	282	1659.6	635	15
79	F	GLA	4.5	143	103	80	n	n	458	2251.6	704	21
63	M	GLA	5	136	95	65	n	n	370	1694.4	618	20
68	M	GLA	19	157	92	79	n	n	338	1978.8	619	21
71	F	GLA	5	173	96	69	n	n	287	1909.5	765	21
24	M	GLA	4.5	162	79	88	y	y	330	1607.5	615	27
82	F	GLA	1.5	142	63	94	y	n	356	2121.4	421	24
86	M	GLA	5	148	89	82	n	n	486	1925.1	627	56
53	F	CT	6	116	67	70	n	n	349	1218.1	679	9
80	F	GLA	4	131	66	74	n	n	325	1735.6	680	33
88	M	GLA	2	132	75	56	n	n	432	2317.8	599	17
75	F	GLA	3.5	169	91	87	n	n	412	2241.8	593	16
77	M	GLA	4	122	60	96	y	y	348	2007.3	628	29
71	F	GLA	5	202	85	68	n	n	353	2146.3	598	13
83	M	GLA	4	153	89	105	n	n	401	1507.6	328	35
57	M	GLA	6	131	81	75	n	n	376	1154.6	586	27
77	M	GLA	4	117	80	84	n	y	435	1911.6	516	18
58	M	CT	18	101	56	71	n	y	415	2397.5	794	11
87	M	GLA	2	140	99	78	n	n	201	1899.1	513	16
88	F	GLA	4	149	48	57	n	n	421	1898.4	592	22
72	F	GLA	3.5	136	66	92	n	n	365	2074.9	562	22
82	F	GLA	3	107	74	112	n	n	359	2074.9	500	43
83	M	GLA	5	131	61	66	n	n	370	1898.4	414	35
72	F	GLA	4	151	85	82	y	n	314	1806.4	691	27
80	M	GLA	3	122	74	64	n	n	422	1804.2	538	21
72	M	GLA	3	178	94	85	n	n	367	1620.2	590	27
80	M	GLA	4	140	80	73	n	n	374	2012.5	498	24
65	M	GLA	2	143	83	73	y	y	311	2135.2	594	33
87	F	GLA	5	148	79	83	n	n	318	1928.4	544	51
88	M	GLA	4	106	55	83	n	n	318	1803.8	458	22
71	M	GLA	5	134	69	68	y	n	322	1803.0	574	26
77	M	GLA	5	118	78	83	n	n	219	1962.7	598	15
68	F	GLA	6	120	78	78	n	n	363	2308.4	646	14
69	M	GLA	4.5	152	72	88	n	n	356	2167.7	676	21
89	F	GLA	5.5	122	80	104	n	n	390	1895.4	537	20
25	F	GLA	5	117	63	62	n	n	555	2289.1	638	18
71	M	GLA	5	144	77	72	n	n	329	1298.8	549	17
83	M	GLA	5	161	90	88	n	n	520	2008.0	288	18
23	M	CT	2	139	89	96	n	n	293	2141.6	824	16
73	M	CT	3.5	179	78	60	n	n	298	2138.3	611	17
58	M	GLA	3.5	128	81	73	n	n	243	2141.9	678	15
75	F	GLA	6	163	87	58	y	n	439	1812.2	567	29
58	F	GLA	5.5	89	62	82	n		445	1959.7	542	21
77	M	GLA	5	160	109	85	n	y	373	1956.2	659	29
68	M	GLA	4	132	68	98	y	y	285	1841.1	736	20
67	M	GLA	4	126	76	80	n	n	387	1631.2	507	22
79	M	GLA	6	121	60	76	y	n	321	1607.2	470	19
80	M	GLA	7	130	68	63	n	n	317	1598.7	511	39
59	M	CT	7.5	166	77	69	n	n	345	1974.8	625	18
78	F	GLA	5	163	65	76	n	n	420	2164.2	661	50
58	F	GLA	3.5	126	71	79	n	n	325	2131.1	668	25
62	F	GLA	7	121	75	83	n	n	423	2137.9	706	14
57	M	GLA	2.5	120	89	91	y	n	346	1905.0	733	23
69	M	CT	4	147	87	74	n	n	346	2041.2	705	13
60	M	GLA	2.5	153	94	96	n	n	383	1893.7	608	29
79	M	GLA	2	122	69	76	y	n	297	1777.7	589	44
81	F	GLA	2.5	175	66	76	n	n	382	2094.3	707	18
70	M	GLA	3	140	79	77	n	n	290	1903.8	635	41
71	F	GLA	4	122	77	75	n	n	409	2163.1	595	28
77	M	GLA	3	168	81	72	n	n	417	1979.0	682	50
68	M	GLA	8	178	70	68	n	n	282	2396.1	517	16
65	F	CT	3	133	78	91	y	n	334	2362.6	830	13.7
84	M	GLA	3	128	70	68	n	n	330	1518.3	615	20
74	F	GLA	1.5	146	82	86	n	n	362	1711.2	757	23
67	M	GLA	1.5	120	64	66	n	n	287	1827.4	615	27
84	F	GLA	4	175	81	98	n	n	380	1845.3	557	36

56	F	CT	8	111	61	80	n	n	310	2077.7	714	16
73	F	GLA	3	126	62	58	n	n	277	1675.8	816	14
59	F	GLA	3.5	156	90	42	n	n	450	1550.8	760	16
88	M	GLA	3.5	191	87	69	n	n	333	1835.6	644	36
59	F	GLA	6	152	80	76	y	n	403	1488.7	864	16
74	F	CT	3	168	98	68	n	n	317	2066.5	727	14
85	M	GLA	7.5	119	71	90	n	n	380	1831.5	574	18
80	F	GLA	7.5	143	75	66	n	n	371	1830.3	514	13
80	M	GLA	0.5	132	57	55	n	n	272	1842.7	453	30
80	M	GLA	3.5	138	87	76	n	n	382	2097.3	688	40
66	F	GLA	2.5	129	70	59	n	n	299	2001.3	794	24
61	F	GLA	2	149	70	78	y	n	366	1786.0	707	22
67	F	CT	2	156	68	67	n	n	375	2001.7	655	16
82	M	GLA	3	120	74	58	n	y	326	1587.3	589	30
75	M	CT	3	170	77	68	y	n	341	2124.1	720	17
80	F	GLA	2.5	150	84	84	n	n	377	2103.1	543	15
84	F	GLA	2.5	133	70	66	y	n	491	2424.8	473	18
75	F	GLA	2.5	111	79	77	n	n	357	1903.7	659	23
83	F	GLA	3	116	72	67	n	n	413	2063.1	525	20
80	F	CT	3	135	83	75	y	n	496	1965.1	629	14
80	F	GLA	3	152	67	77	n	n	418	1775.7	600	23
78	M	GLA	3	140	74	75	y	n	337	1624.8	458	16
67	F	GLA	3	130	63	66	n	n	315	1944.6	538	24
73	F	GLA	3	179	80	53	y	n	319	2099.7	417	27
77	F	CT	4	121	70	60	n	n	376	2033.1	376	16
83	M	GLA	1.5	124	47	101	y	n	485	1885.0	628	21
65	M	GLA	2.5	109	71	66	y	n	257	1867.7	561	17
66	M	GLA	2.5	138	78	69	n	n	297	2043.6	672	33
74	M	GLA	7	166	97	64	n	n	427	1995.4	615	20
72	M	GLA	5	152	99	79	y	n	320	1959.2	731	19
71	M	GLA	3.5	147	88	100	n	n	266	1843.1	741	16
76	F	GLA	3.5	129	57	81	n	n	366	2286.3	591	24.1
73	F	GLA	3	172	87	72	y	n	349	2146.3	721	18
73	F	CT	3	126	71	106	n	n	426	2111.9	710	16
79	F	CT	3	131	69	64	n	n	354	2021.7	572	12
61	F	GLA	3	138	88	64	n	n	453	1746.9	678	19
71	F	CT	3	147	72	95	n	n	431	1995.3	524	17
80	F	GLA	3	130	76	65	n	n	338	2274.6	628	22
92	F	CT	3	117	50	71	n	y	363	2202.7	521	17
79	M	GLA	2.5	147	76	59	n	n	380	2285.5	710	58
77	F	GLA	3	162	83	62	y	n	395	2102.2	659	24
83	M	GLA	3.5	147	88	66	n	n	411	2199	637	31
76	F	GLA	4	145	62	61	n	n	338	2105.1	680	17
78	M	GLA	2.5	159	77	72	n	n	392	2504.5	646	16
75	F	GLA	4	158	82	91	n	n	346	2233.6	726	19
70	F	CT	4	147	76	62	n	n	430	2113.4	653	19
84	F	GLA	3.5	142	86	70	n	n	359	2194.9	589	18
58	M	GLA	4	150	88	62	n	n	282	1939.0	884	20
86	F	GLA	4	139	61	90	n	n	506	2053.6	736	22
76	M	GLA	2	163	76	70	n	y	339	1707.1	713	20
72	F	GLA	2	144	69	80	n	n	308	2078.3	567	25
70	F	CT	2	110	73	70	n	n	347	1804.5	734	19
69	M	CT	3	126	64	69	y	y	334	1919.6	545	18
72	F	CT	3	134	63	63	n	n	318	1676.2	624	17
77	F	GLA	2.5	159	48	79	n	n	464	2120.4	588	22
77	M	GLA	3	174	109	86	n	n	414	1962.9	660	38
68	F	GLA	3.5	140	75	67	n	n	473	2041.5	577	21
79	F	CT	2.5	116	60	70	n	n	370	2013.7	601	11
84	F	GLA	3.5	142	56	66	n	n	337	1929.4	560	15
74	M	GLA	2	105	59	55	y	n	403	1944.1	331	14
79	F	GLA	2.5	148	77	58	n	n	380	1909.1	628	17
85	F	GLA	3	131	76	94	n	n	374	1762.1	551	16
77	F	CT	3	127	78	66	n	n	384	2110.7	529	16
78	F	CT	3.5	152	79	79	n	n	395	2509.8	590	20
61	M	GLA	3	132	52	52	n	n	399	1685.2	598	40
90	F	GLA	4	154	81	81	n	n	501	2172.8	422	20
69	M	GLA	5.5	155	93	70	n	n	304	1296.8	820	18
80	F	GLA	2.5	153	75	76	n	n	335	1870.6	726	23
66	F	CT	3	124	63	78	y	n	354	1868.7	712	18
70	F	CT	3	134	63	65	y	n	290	1693.8	699	15
82	F	GLA	3	140	75	83	n	n	361	2079.5	544	19
77	F	GLA	1.5	132	81	74	n	n	318	2121.1	551	17
69	F	GLA	2.5	120	61	68	n	n	328	1984.5	660	27
78	F	GLA	3	137	75	72	n	n	325	2118.6	576	30
77	F	GLA	3	138	61	83	n	n	426	2048.6	428	19
85	F	GLA	3	158	76	58	n	n	350	1924.6	527	20
96	F	GLA	3	158	76	58	n	n	242	2068.7	607	18
67	M	GLA	2.5	176	93	80	y	y	343	1945.3	513	15
85	F	GLA	3	132	65	62	n	n	415	1708.8	578	25
78	F	CT	3.5	129	76	84	n	n	282	1761.0	558	20
80	M	GLA	3	85	51	83	n	n	394	1854.2	554	23
86	M	GLA	4	115	80	74	n	n	379	2269.7	513	30
73	F	GLA	3.5	103	51	48	n	y	262	1806.2	628	42
72	M	GLA	1	144	79	78	n	n	397	1724.4	619	24
83	F	GLA	3	154	81	67	n	n	313	1916.2	590	18
74	F	CT	3	163	98	93	n	n	430	2058.5	713	16
65	F	CT	3	116	71	69	n	n	401	2078.8	592	17
77	F	CT	3	117	70	66	n	n	355	1730.9	635	12
87	M	GLA	2.5	127	66	71	n	n	202	2032.2	507	18
85	F	GLA	3	119	61	69	n	n	406	1889.5	537	21
82	M	CT	2.5	113	72	68	y	n	337	1850.2	557	19
86	F	GLA	3	138	74	72	n	n	430	2021.0	558	20
78	M	GLA	3	132	74	68	n	n	307	2037.5	653	33
72	F	CT	3	136	82	52	n	n	352	1979.3	645	19
76	F	GLA	3	131	74	78	n	n	498	2187.0	534	15
78	F	GLA	4	141	80	79	n	n	365	2039.5	542	16
85	M	GLA	4	155	56	74	n	n	420	2298.2	572	17
88	M	GLA	3.5	123	69	73	y	n	260	2070.5	470	12
61	F	GLA	3.5	113	69	79	n	n	355	1805.8	643	16
53	M	GLA	12	126	78	69	y	y	488	2193.9	671	36
79	M	GLA	5	120	68	91	y	n	346	2002.5	589	23
61	F	CT	3	102	56	64	n	n	333	1918.1	580	12

76	F	CT	3	155	86	80	n	n	410	2175.4	562	12
74	F	CT	3.5	116	67	74	y	n	511	2115.4	501	12
71	F	CT	3	112	62	59	n	n	290	1873.8	585	17
65	F	CT	3	158	80	60	n	n	435	2074.6	606	19
78	F	GLA	4	132	65	61	n	n	368	1820.5	554	20
73	F	GLA	3.5	135	57	54	y	n	430	1918.5	564	19
83	F	CT	3	142	51	58	n	n	362	2113.1	505	11
77	F	GLA	3	105	67	70	y	n	303	1732.5	490	17
55	M	GLA	4	123	80	84	n	n	435	2037.6	677	19
90	F	CT	4	131	70	68	y	n	407	2126.8	604	12
86	M	GLA	4.5	150	60	58	y	n	400	2042.6	633	15
61	M	CT	4.5	117	63	46	n	y	327	2118.2	695	19
79	F	CT	4.5	144	79	65	n	n	226	2030.2	624	18
84	F	GLA	2.5	158	77	82	n	n	270	2143.4	624	25
77	M	GLA	4.5	116	79	82	n	y	448	2017.4	792	18
90	M	GLA	2	144	81	81	y	n	372	1903.1	615	44
62	M	GLA	3.5	140	79	67	n	n	328	2094.4	753	20
60	M	GLA	4.5	130	87	85	y	n	258	1958.7	629	42
83	F	GLA	3	114	51	79	n	n	305	1682.2	786	19
83	F	GLA	3	134	81	87	n	n	522	1974.9	812	19
81	M	GLA	2	146	58	51	n	n	311	2513.6	693	17
81	F	CT	2.5	169	59	82	n	n	292	2214.2	692	19
83	F	GLA	2	137	56	66	n	n	337	2365.7	690	14
74	M	GLA	2	128	80	73	n	n	352	2309.7	721	21
64	M	GLA	2.5	138	76	75	n	n	251	2338.7	697	16
76	F	GLA	2	126	71	62	n	n	458	2285.6	704	16
77	F	GLA	3	128	70	74	n	n	380	2162.8	738	21
73	M	CT	2	149	72	62	y	n	414	2041.5	686	16
68	F	CT	4.5	155	81	67	n	n	415	2191.7	775	18
63	M	CT	4	125	75	74	n	n	334	1946.3	805	14
88	F	CT	3	131	62	64	y	n	407	2125.2	583	16
66	F	CT	4.5	117	66	70	n	n	439	1855.5	676	17
80	M	CT	4.5	113	83	76	n	n	375	1556.3	576	18
39	M	CT	16	146	85	105	n	y	319	2055.4	798	15
55	F	CT	6	126	76	75	n	n	307	1835.6	617	17
92	M	GLA	2.5	157	90	93	n	n	308	1934.8	710	31
53	F	GLA	3	155	96	76	n	n	438	2040.3	806	56
80	M	GLA	2.5	132	62	71	y	n	365	1511.3	693	18
57	M	GLA	4	154	78	61	n	n	313	1968.2	748	23
76	M	GLA	14	170	78	53	n	y	283	1878.9	833	41
80	F	CT	2	147	78	95	n	n	405	2060.4	678	20
61	F	GLA	3	111	69	73	n	n	354	1892.4	734	15
58	F	CT	1	140	83	68	n	n	324	2069.4	704	20
71	F	CT	14	156	84	68	n	n	328	1926.5	712	19
89	F	CT	2	140	45	57	n	n	286	1947.5	561	15
78	F	CT	2.5	96	62	62	n	n	291	1828.4	631	12
78	F	CT	3	161	83	60	y	n	323	1818.7	374	16
64	F	CT	2.5	118	64	67	n	n	361	2001.5	643	14
66	F	CT	3	108	53	62	n	n	337	1808.4	507	18
76	F	CT	4	149	70	51	n	n	335	1966.1	670	17
80	F	GLA	3	155	77	72	n	n	470	1981.2	695	15
91	F	CT	2.5	148	79	63	n	n	376	1866.0	482	19
82	M	CT	3	149	63	59	n	n	358	1934.8	544	17
72	F	CT	3	126	65	74	n	n	327	1871.3	675	17
72	F	GLA	3	154	84	61	n	n	318	1896.4	734	19
84	F	GLA	2	124	63	76	n	n	306	2020.6	662	15
85	M	GLA	2.5	119	57	72	n	n	421	1924.5	521	27
74	F	GLA	4	188	100	72	n	n	414	2014.1	682	18
82	F	GLA	3.5	118	72	66	n	n	366	1850.0	653	21
74	M	CT	3	149	74	74	n	n	332	1829.0	626	14
69	F	CT	4	127	79	77	n	n	345	1753.0	713	17
79	F	GLA	3	158	73	59	n	n	306	1925.3	522	23
73	F	CT	3	123	63	79	n	n	354	2164.2	770	20
84	F	GLA	3	147	61	75	y	n	310	1848.0	594	50
83	F	GLA	2	121	65	88	n	n	368	1708.7	572	42
81	M	CT	2	165	90	82	y	n	347	2015.2	645	19
81	F	CT	3	123	64	87	n	n	364	1947.3	513	16
80	F	GLA	2	136	60	88	y	n	313	1863.5	633	25
87	M	GLA	2.5	155	77	65	n	n	319	1893.3	561	39
83	F	GLA	4.5	154	82	64	n	n	465	2016.7	496	24
80	M	GLA	2	168	72	58	n	n	400	1517.2	430	36
84	M	GLA	2.5	101	61	75	n	n	403	2287.3	557	34
71	M	CT	2	130	74	77	y	n	219	2256.4	640	13
80	F	GLA	2.5	149	73	59	n	n	332	2722.6	652	14
90	M	CT	3	131	75	86	n	n	374	2622.9	525	15
70	F	GLA	3.5	135	75	58	n	n	360	2857	646	19
76	F	CT	8.5	126	61	83	n	n	311	1836.4	576	17
81	F	CT	3	96	61	78	n	n	354	2064.9	455	16
81	F	GLA	3.5	145	71	67	n	n	268	1725.2	635	22
79	F	GLA	4	151	89	114	n	n	436	2056.3	565	17
73	M	CT	4	136	81	57	n	n	284	1888	559	10
76	F	GLA	3.5	134	83	55	n	n	271	2162.1	607	18
78	F	GLA	4.5	148	88	70	n	n	368	1858.3	565	22
81	M	CT	5	137	70	59	n	n	222	2813.2	510	17
78	M	GLA	5	143	83	69	n	n	326	2359.4	366	17
76	F	GLA	2.5	156	75	91	n	n	364	1920.3	601	42
54	M	GLA	6	106	60	65	n	y	303	1654.8	750	18
71	F	GLA	7	128	88	57	n	n	338	1764.7	662	19
77	M	GLA	3.5	111	53	67	n	n	202	2074.4	747	32
80	M	GLA	2	150	65	62	n	n	204	1833.0	660	11
74	F	CT	2	139	67	60	n	n	317	2027.6	613	16
75	F	CT	2	130	70	79	n	n	405	2022.6	621	16
77	F	GLA	2.5	138	78	78	n	n	402	1680.2	622	15
73	F	GLA	2.5	138	73	62	n	n	340	1787.8	697	43
70	F	GLA	3.5	133	77	82	n	n	380	2095.9	677	21
66	F	GLA	2.5	111	60	78	n	n	230	2070.5	680	16
76	F	GLA	2	148	70	72	n	n	340	1972.6	669	18
74	F	CT	2	144	76	88	n	n	261	2038.3	611	20
90	F	GLA	3	159	87	58	n	n	297	2304.0	607	18
69	F	GLA	3	123	72	64	n	n	302	1957.3	675	20
79	F	CT	3	134	80	88	n	n	502	1956.3	548	15
77	F	CT	2.5	156	64	67	n	n	357	1810.0	624	15

69	M	CT	4	144	67	68	y	y	232	1696.2	701	20
75	F	GLA	3.5	129	63	59	n	n	374	1567.9	485	17
88	F	GLA	3	112	66	60	n	n	349	1815.8	565	20
82	F	GLA	3.5	134	71	78	n	y	262	2197.0	523	19
68	M	GLA	4	127	72	57	n	n	334	1967.1	534	18
75	F	GLA	3.5	172	85	72	n	n	379	1241.5	674	17
79	F	GLA	5.5	158	70	64	n	n	387	2042.6	516	17
61	F	CT	5	117	69	73	n	n	468	2341.7	604	18
79	F	GLA	5.5	151	91	81	n	n	408	1955.2	599	14
49	F	GLA	4	160	102	89	n	n	314	2012.7	663	25
85	M	GLA	3	119	60	63	n	n	286	1414.3	499	20
81	M	GLA	5	142	82	77	n	n	312	2020.8	574	16
82	M	GLA	1	159	65	59	n	n	346	2020.2	609	48
73	F	CT	2.5	117	58	63	n	n	298	1823.5	513	17
63	F	CT	2.5	164	82	54	n	n	258	1756.9	692	16
74	F	GLA	2	118	73	71	n	n	246	1950.2	627	20
74	F	CT	2	160	84	91	n	n	305	2031.3	717	20
86	M	GLA	3.5	160	77	60	n	n	292	1760.1	513	45
71	F	GLA	3.5	90	53	62	n	n	346	2122.8	551	12
77	F	GLA	4.5	158	66	62	y	n	467	1870.9	545	28
71	F	GLA	4.5	121	64	55	n	n	340	1792.2	587	17
84	M	GLA	3.5	153	63	71	n	y	372	2205.3	457	34
87	M	GLA	7	127	80	66	n	n	386	2520.9	503	21
65	M	CT	1	118	67	71	n	y	456	1894.6	537	20
75	F	GLA	3	162	80	58	y	n	322	2009.1	623	14
78	M	GLA	2.5	144	84	69	n	n	342	1931.0	589	25
82	M	GLA	3	85	55	67	y	n	476	2476.4	395	13
73	F	GLA	3.5	154	77	63	n	n	363	2035.0	620	19
65	F	CT	3.5	135	81	67	n	n	344	1523.4	621	13
80	F	GLA	3.5	181	66	66	n	n	309	1911.3	558	16
74	F	CT	3.5	162	73	66	n	n	409	2057.9	625	12
75	F	GLA	2	111	62	89	y	n	410	1959.4	516	17
80	M	GLA	4	135	70	78	y	n	299	1642.4	624	25
83	F	GLA	5	140	83	77	y	n	419	2116.0	453	18
63	M	GLA	3.5	180	100	78	y	n	453	2213.0	605	13
55	F	GLA	3.5	123	80	72	n	n	461	2057.0	624	22
70	F	GLA	4	145	80	71	n	n	358	2031.7	609	21
81	M	GLA	4	159	67	59	n	n	343	1321.3	611	21
80	F	GLA	5.5	115	68	70	n	n	397	1814.7	531	14
84	M	GLA	4.5	110	58	78	n	n	402	2255.6	699	26
66	M	GLA	5.5	160	87	64	y	n	387	1865.4	705	22
71	M	GLA	2.5	133	71	88	y	n	300	1977.8	575	17
78	M	GLA	3	136	68	95	n	n	348	1756.7	556	41
81	M	GLA	2.5	134	73	73	y	n	335	2224.1	600	21
76	F	GLA	3.5	135	67	69	y	n	284	2030.2	591	26
84	F	GLA	3.5	167	97	79	n	n	385	2228.5	562	16
77	F	CT	4	130	69	71	n	n	346	2502.7	576	17
82	M	GLA	3.5	130	65	58	n	n	393	2114.4	708	14
72	M	CT	4	144	75	73	n	n	339	1846.5	633	14
87	M	GLA	4	146	55	55	n	n	380	1473.1	619	17
71	F	CT	6	142	74	66	n	n	361	2037.3	526	15
64	F	GLA	3.5	169	94	70	n	n	401	1969.9	521	44
84	M	GLA	6	128	83	69	n	n	452	2074.7	734	38
84	F	GLA	2.5	124	66	87	y	n	403	2070.4	659	20
67	M	GLA	3.5	123	78	77	y	n	393	2047.7	764	27
76	M	GLA	5.5	159	93	99	n	n	489	2281.2	552	64
78	F	GLA	3	132	81	78	y	n	416	1959.9	570	23
79	F	CT	2	97	57	56	y	n	305	2220.0	579	16
71	F	CT	2.5	129	83	57	n	n	313	2100.6	572	20
69	F	CT	3.5	115	69	74	n	n	422	2109.7	584	18
51	M	CT	4.5	144	87	53	n	n	362	1886.2	675	16
79	F	GLA	4	126	66	91	n	n	316	1879.1	433	10
66	F	GLA	6.5	181	108	58	n	n	357	1416.6	550	40
76	F	GLA	6	171	94	69	y	n	363	2119.0	545	22
73	F	GLA	3	142	72	68	n	n	413	1943.4	545	13
89	F	GLA	4	141	61	74	n	n	374	1964.9	500	11
81	F	GLA	4	134	64	86	n	n	345	1364.7	529	18
78	M	GLA	4	158	67	64	y	n	312	1818.1	606	15
65	F	GLA	3.5	165	83	52	n	n	469	2259.1	612	27
80	M	GLA	4.5	148	77	76	y	n	249	1874.9	534	38
69	M	GLA	2.5	164	99	73	y	n	318	1958.5	592	25
93	M	GLA	2	167	64	57	n	n	434	2108.1	257	15
86	F	CT	2	134	65	76	n	n	370	2128.1	486	11
83	M	GLA	3	162	70	51	n	n	374	2030.5	580	17
77	F	CT	2	148	87	82	n	n	323	2173.2	519	18
74	F	GLA	5	120	69	65	n	n	346	1926.8	475	19
80	M	GLA	3	134	76	71	n	n	291	1841.8	552	33
51	F	CT	1	158	90	86	n	n	372	1627.6	596	14
36	F	CT	1	94	59	88	n	y	327	2239.2	631	14.7
70	F	GLA	3	102	62	60	n	n	359	1847.6	477	22
67	F	GLA	4.5	137	69	66	n	n	369	1539.8	523	25
73	M	GLA	4	125	64	71	n	n	325	1998.6	509	18
73	F	GLA	2	139	83	80	n	n	341	1742.2	591	22
86	M	GLA	4.5	192	84	62	n	n	447	2339.3	486	14
80	M	GLA	3	145	59	54	n	n	276	2103.7	455	35
85	F	GLA	4	166	57	57	n	n	397	2120.2	474	13
82	F	CT	7	121	67	57	n	n	330	2638.6	495	14
81	M	GLA	5.5	142	77	81	y	n	364	2024.4	506	30
62	F	GLA	6	124	64	80	n	n	305	2139.9	646	14
82	M	GLA	2	148	89	74	n	n	413	2089.4	574	14
75	F	GLA	3.5	135	86	96	n	n	424	2067.7	554	25
70	F	GLA	3.5	157	77	65	y	n	380	1750.1	591	17
76	F	CT	4.5	134	80	74	y	n	357	2658.0	551	17
54	M	CT	3.5	145	85	79	n	n	329	1888.8	689	18
79	F	GLA	6.5	113	76	75	n	n	428	1985.3	564	21
72	F	GLA	3	169	79	62	n	n	298	2491.5	624	17
53	F	GLA	3.5	97	61	83	y	n	423	2078.5	677	24
64	M	GLA	3	147	89	61	n	y	351	2528.0	657	13
61	F	GLA	3	116	69	70	n	n	516	2505.2	602	40
78	F	CT	2.5	145	84	73	n	n	322	2093.9	595	18
86	F	GLA	3.5	140	75	89	n	n	412	2340.6	451	19
77	F	GLA	5	122	58	79	y	n	403	2063.4	608	22

56	M	GLA	2.5	133	90	86	n	y	420	1991.6	550	26
92	M	GLA	2.5	146	66	82	n	n	262	2079.2	547	15
66	M	GLA	4	126	72	88	n	n	308	2064.4	625	17
77	F	GLA	3.5	153	70	54	n	n	362	2143.8	548	10
57	M	GLA	4.5	144	81	53	n	y	353	1571.1	602	16
64	F	GLA	2	131	62	82	n	n	348	2189.6	554	21
78	M	GLA	5	130	72	64	n	n	213	1801.1	600	34
48	F	GLA	3.5	147	98	89	n	n	296	2152.6	618	23
84	M	GLA	5	140	76	66	n	n	397	2664.6	526	13
79	F	GLA	3	149	70	74	n	n	267	2030.4	630	12
90	M	GLA	4	176	95	76	n	n	326	1699.1	512	72
74	M	GLA	3	149	81	73	n	y	246	2303.6	604	30
61	F	GLA	2.5	120	71	76	n	n	298	1650.7	616	18
50	M	GLA	5	173	119	79	n	n	299	1532.9	651	44
78	F	GLA	3.5	151	123	55	n	n	437	2426.2	636	25
82	F	GLA	2.4	114	55	69	n	n	423	2486.9	608	58
80	M	GLA	2	94	46	71	n	y	295	2180.1	578	18
67	M	CT	4.5	151	75	69	n	n	301	2137.1	645	17
71	F	GLA	3	136	70	88	y	n	354	2301.0	644	23
77	M	GLA	6	122	64	72	n	n	362	2367.4	493	16
76	M	GLA	15	139	71	75	y	n	315	2316.4	607	26
78	F	GLA	3.5	139	88	77	n	n	302	2142.7	615	20
90	M	GLA	3.5	118	88	70	n	y	350	1940.7	482	21
75	F	GLA	4	128	89	67	n	n	402	1971.9	526	40
90	M	GLA	2.5	148	88	90	n	n	404	2306.8	601	34
63	M	CT	4.5	132	93	79	n	y	422	2160.8	652	16.3
86	F	GLA	4	157	88	88	n	n	281	1991.8	639	23
63	M	CT	4	141	94	109	n	y	314	1938.0	738	14
74	F	GLA	5	146	73	63	n	n	387	1692.6	496	18
80	M	GLA	5.5	120	72	70	n	n	292	1982.0	539	42
76	M	GLA	6.5	144	79	73	n	n	352	1342.8	538	25
81	M	GLA	6	133	79	95	n	n	232	2296.9	463	37
70	F	GLA	3	114	69	75	n	n	311	2066.9	528	30
82	F	GLA	6.5	135	83	89	n	n	281	2141.3	489	21
